# Improving mental health literacy of frontline community health workers in a rural district of Pakistan: mPareshan project

**DOI:** 10.1192/bjo.2026.11051

**Published:** 2026-05-25

**Authors:** Samina Akhtar, Fauziah Rabbani, Javeria Nafis, Amna Siddiqui, Zul Merali

**Affiliations:** Department of Community Health Sciences, The Aga Khan University, Pakistan; Brain and Mind Institute, The Aga Khan University, Pakistan

**Keywords:** Mental health, frontline workers, training, mhGAP, knowledge and skills

## Abstract

**Background:**

In low- and middle-income countries, four out of five people with mental illness do not receive specialised treatment. Utilising non-specialist frontline workers to deliver basic mental health services at the community level therefore warrants exploration.

**Aims:**

This study assessed improvement in the knowledge and skills of frontline community workers in identifying symptoms of anxiety and depression, making appropriate referrals and providing psychosocial counselling, in a rural district of Pakistan.

**Method:**

Project mPareshan developed a training manual to enhance the mental health literacy of government-employed lady health workers (LHWs) and lady health supervisors (LHSs). Content was adapted from the World Health Organization’s Mental Health Gap Action Programme 2.0 intervention guide to suit the local context. A total of 72 participants (36 LHSs and 36 LHWs) from the Badin District, Sindh, Pakistan, received the training. Pre- and post-tests were conducted to assess changes in knowledge and skills, using the Wilcoxon signed-rank test.

**Results:**

There was a statistically significant improvement in both knowledge (*p* < 0.01, *r* = 0.85) and competency (*p* < 0.01, *r* = 0.81) median scores following the mPareshan training. LHSs demonstrated higher percentage increase in knowledge and competencies in domains requiring practical application, such as coping mechanisms, psychosocial support and referral pathways, compared with LHWs, highlighting the importance of their supervisory role and support in mental health service delivery.

**Conclusions:**

The mPareshan mental health training has the potential to improve the knowledge and competencies of community health workers. Such initiatives can be scaled up to enable frontline workers to function as an effective workforce in the absence of specialist mental health services.

In 2021, around 14% of the world’s population experienced mental health disorders. Out of these, depression and anxiety were the most prevalent and are estimated to have caused 46 and 43 million disability-adjusted life-years, respectively.^1^ In Pakistan, studies over the past decade estimate the burden of depression and anxiety between 22 and 60%.^2–4^ The vast majority – over 75% – of the treatment gap for mental disorders exists in low- and middle-income countries (LMICs), where four out of five individuals with mental illness do not receive effective treatment.^5^ A major barrier in addressing this gap in LMICs is the limited availability and unequal distribution of specialist mental health professionals.^6^ As a result, a significant proportion of individuals who would benefit from care remain untreated.^7^ Although integration of mental health into primary care is an established global practice,^8^ Pakistan faces a significant implementation gap because of a scarcity of trained non-specialist personnel.^9^ With approximately 110 000 community health workers (CHWs) embedded in communities across the country, there exists a unique opportunity to expand access to basic mental healthcare through this existing primary healthcare workforce.

## The Mental Health Gap Action Programme

To address these global and national priorities, the World Health Organization (WHO) has developed a set of tools, including the Mental Health Gap Action Programme Intervention Guide (mhGAP-IG), to support the assessment and management of priority mental health disorders in non-specialised settings.^10^ Following the first publication of the mhGAP-IG in 2010, a revised version (mhGAP-IG 2.0) was released in 2016, and the WHO plans to provide regular updates to align with emerging evidence.^11^ In 2019, the mhGAP Community Toolkit was launched for field testing.^12^

The mhGAP-IG 2.0 training guide includes information for programme managers and a practical manual for those promoting and supporting community-level mental health interventions.^10^ In several African contexts like Nigeria, Cameroon, Tanzania and Ethiopia, mhGAP training has been conducted to increase capacity of primary healthcare staff (including physicians, clinical officers, nurses, psychologists and community health extension staff with heterogenous levels of education and mental health expertise) to improve case identification, referrals and psychoeducation skills.^13–15^ In Mozambique, mhGAP materials were adapted for training a broader group of CHWs, including leaders, traditional and faith healers, and teachers – leading to increased referrals and improved help-seeking behaviour.^16^ Studies in South American countries like Chile and Peru also report similar positive uptake of training in primary healthcare workers.^14^

## Pakistan’s Lady Health Worker Programme

In Pakistan, CHWs, which comprise lady health workers (LHWs) and lady health supervisors (LHSs), serve their rural communities and urban slums, as the outreach extension arm of primary healthcare providing preventive and promotive maternal and childcare at the household level.^17^ LHWs have barely 8 years of formal education and undergo 18 months of structured on-job training. Their existing curriculum lacks a dedicated mental health component despite their close engagement with communities experiencing psychological distress. Their embeddedness within the community allows them to navigate local customs, languages and social dynamics more effectively than external health providers.^18^

## Project mPareshan

Against this backdrop, we implemented project mPareshan,^19^ a home-based mental health intervention to reduce anxiety and depression through frontline LHWs in rural Sindh, Pakistan. This paper reports on the development and evaluation of a locally adapted mhGAP-IG 2.0 based training as part of the project, assessing its impact on mental health-related knowledge and skills among LHWs and LHSs. In contract to several other settings, the novelty of this study lies in adapting the mhGAP-IG for a low literacy, non-specialist female workforce with no prior mental health training or background. These LHWs only provide care at community doorsteps and are not affiliated as service providers with any formal healthcare facility. Therefore, the research question we explore is: can a tailored mhGAP training programme enhance mental health knowledge, competencies and practices among women lay health workers (LHWs/LHSs) with limited literacy and no mental health training, operating in rural Pakistan’s community-based primary healthcare system? By contextualising the manual and assessing change in knowledge and skills post training, we demonstrate the feasibility of scaling a culturally relevant mhGAP approach through Pakistan’s primary care infrastructure.

## Method

In collaboration with the LHW Programme (LHW-P) and Department of Health Government of Sindh, Project mPareshan was implemented in Badin, a rural coastal district, which has a population of about 1.8 million.^19^ The literacy rate is 24%,^19^ with two-thirds of the households in severe poverty.^20^ Badin reports one of the highest suicide rates in the province and has limited healthcare infrastructure. The LHW-P in Badin roughly includes 1100 LHWs supervised by 36 LHSs.^19,21^

Each LHW covers a catchment area of 1000 people on average, in their community. A single LHS oversees the work of 25–30 LHWs, conducts routine supervisory visits, and participates in monthly review meetings to discuss health progress and issues with LHWs. With about the same level of education, LHSs are typically senior in terms of their work experience compared with LHWs.^18,22^

### Development of the mental health training manual for CHWs

The lack of culturally sensitive training materials hampers the effective transfer of mental health knowledge and skills, limiting uptake and application among non-specialist workers. This highlights a pressing need for tailored resources that cater to the linguistic and cultural nuances of the local population. Therefore, as part of the mPareshan project, a comprehensive literature review and analysis of stakeholders’ (health workers, LHW-P managers, community and policy makers) perspectives was carried out. This review highlighted gaps in LHWs’ knowledge and skills in recognising and managing common mental health disorders, particularly anxiety and depression. mhGAP-IG 2.0 was therefore adapted with a focus on cultural relevance and acceptability. The adapted manual was translated into both national (Urdu) and provincial (Sindhi) languages for linguistic alignment with LHWs’ and LHSs’ literacy levels. The manual incorporated pictorial sketches and scenarios that reflected local Sindhi culture, enhancing the relevance and engagement of LHWs in psychosocial counselling as part of their routine work.

The adapted manual^23^ comprised four modules: Introduction, Essential Care and Practice, Anxiety and Depression, and Counselling Strategies. An overview of the training content is given in Table 1. Key changes included using local expressions for anxiety and depression, simplified language that was easily comprehensible, excluding clinical diagnostics and pharmacological management content, and adding context-specific terms like LHWs/LHSs in lieu of ‘healthcare providers’. Other key replacements included adopting the term ‘counselling strategies’ instead of ‘management of MNS (mental, neurological, and substance use) disorders’ and clumping self-harm/suicide risk categories as danger signs prompting immediate referral. The manual aligned referral pathways with nearby healthcare facilities and private practitioners, addressing the scarcity of mental health specialised services in the region.

**Table 1:** Overview of mPareshan training content

Days	Number	Modules	Objectives By the end of training, an LHW/LHS will be able to;	Duration in min	Session’s outline/methodology
Training day 1	1	Introduction	Develop a basic understanding of mental health Understand the importance of mental health and its burden in Sindh	35	Session 1: Welcome, participants’ introduction and overview of the workshop
				60	Session 2: Pre-test (knowledge and skill assessment)
				25	Session 3: Mental health definition, importance and current situation PowerPoint presentation
	2	Essential care and practice	Understand and practice effective communication skills while interacting with community members suffering from mental health issues Understand how to treat community members experiencing mental health challenges with respect and dignity	60	Session 1: General principles of essential care and practice PowerPoint presentation Group discussion Facilitator demonstration of good versus poor communication skills Practice effective communication skills
Training day 2	3	Anxiety and depression	Understand the definition of anxiety Demonstrate and recognise symptoms of anxiety Recognise the common and core symptoms of depression Demonstrate the key difference between anxiety and depression Understand the causes of anxiety and depression	60	Session 1: Anxiety PowerPoint presentation Activity to learn the use of algorithm
				120	Session 2: Depression PowerPoint presentation Activity to learn the use of algorithm Video demonstration of a person’s story Group discussion Role-plays to identify symptoms of anxiety or depression
Training day 3	4	Counselling strategies	Understand different ways to decrease the symptoms of anxiety or/and depression Provide counselling to a person with symptoms of anxiety or/and depression Recognise signs of common mental health conditions that require referral to a mental health specialist and make appropriate referral	90	Session 1: Mental health counselling strategies PowerPoint presentation Group discussions Role-plays to practice psychosocial interventions
				30	Session 2: Referral for specialised mental health services PowerPoint presentation Group discussions
				60	Session 3: Post-test

LHW, lady health worker; LHS, lady health supervisor.

Thus, using a contextually relevant approach, the adapted manual aimed to enhance LHWs’ and LHSs’ capacity to recognise, manage and refer mental health cases in primary healthcare settings while also improving their communication and psychosocial counselling skills.

### Training workshops

Two on–site training workshops were conducted in 2022, each lasting 3 days. Training was facilitated by a lead researcher and a co-facilitator, using a blend of pedagogical methods including PowerPoint presentations, video demonstrations and role-play exercises. Modules 1 and 2 were covered on the first day, followed by modules 3 and 4 on days 2 and 3. Participants’ knowledge and skills related to mental health were assessed both pre and post training by using the specified study instruments.

### Study instruments

Knowledge was assessed using a ten-item multiple-choice questionnaire adapted from the mhGAP-IG 2.0. Three items were scenario-based and prompted options, from which participants selected one. The questions assessed knowledge of effective communication techniques, symptoms of anxiety and depression, danger signs, psychosocial counselling and appropriate referral, etc. Each correct answer was awarded 1 point, whereas a wrong answer was scored 0.

Similarly, competency was assessed using a ten-item checklist evaluating effective communication (six items), psychosocial counselling (three items) and appropriate referral decision (one item). These competencies were assessed through role-plays conducted by the training team (lead researcher and co-facilitator). One of them assumed the role of an LHW providing care, and the other portrayed an individual seeking care for mental health issues. Participants of the training evaluated the role-play and scored the simulated interaction on the competency assessment as task performed (score 1) or not performed (score 0).

### Sample size and participant selection

Our sample size was informed by existing studies that used mhGAP training as the mental health intervention and non-specialist CHWs as study population in comparable settings and reporting similar outcomes of change in knowledge and skills scores.^9,24^ Using Open Epi (version 3.01, Emory University, Atlanta, Georgia, USA; https://www.openepi.com/Menu/OE_Menu.htm), we estimated the required sample size based on a mean difference of 1.5 (s.d. = 3.9) in knowledge scores before and after training, as reported in a previous study.^25^ Assuming 5% error of significance and 80% power, the sample size required 72 CHWs, adjusted for 10% drop-out rate. Accordingly, we selected all 36 LHSs and the 36 most active LHWs in Badin for participation in the mPareshan training. An ‘active’ LHW was defined as one who had promptly attended maternal, neonatal and child health (MNCH) programme meetings, conducted regular household visits and submitted monthly reports over the preceding 3 months. This deliberate sampling ensured that the participants of the training had the necessary experience and contextual understanding of their community, and potential to implement the intervention in a real-life setting.

### Data analysis

Effectiveness of the mPareshan training was evaluated by comparing pre- and post-test scores of knowledge and competency among the LHSs and LHWs. The overall knowledge and competency scores were calculated as composite scores of the ten-item questionnaires. Each training participant was assigned a unique code. The data were not normally distributed, as assessed by Shapiro–Wilk test, and because of the ordinal nature of the scores, the Wilcoxon signed-rank test was conducted to compare participants’ median knowledge and competency scores before and after the mPareshan training, using Stata 14.2 for Windows (StataCorp, College Station, Texas, USA; https://www.stata.com/stata14/). Results are reported as medians with interquartile ranges (IQRs), and effect size was calculated as a rank-biserial correlation using the formula *r* = *Z*/√*N*. Item-wise knowledge and competency responses for LHSs and LHWs were reported as number and percentage of participants responding correctly to each item. Results are presented separately for LHSs and LHWs to highlight differences in their knowledge and competency levels.

### Ethics approval and consent to participate

The study received ethical approval from the Aga Khan University’s Ethical Review Committee (approval number 2021-6570-20015). Written informed consent for knowledge and skills assessments was obtained from all participants before starting the training. Names and other identifying information of the participants were masked using a unique participant identifier. Project mPareshan is a registered trial at the Australian New Zealand Clinical Trial Registry (registration number ACTRN12622000989741).

## Results

A total of 72 participants (36 LHWs and 36 LHSs) received the mPareshan training to improve their knowledge and skills related to basic mental health. The median age was 37 years (range: 29–45) for LHSs and 33 years (range 22–55) for LHWs. Median years of experience was 12 in both cadres (range: 8–20), in their current designation with the LHW-P.

### Overall change in knowledge and competency scores

Paired comparisons revealed statistically significant enhancements in healthcare workers’ knowledge and competencies after the training (Wilcoxon signed-rank test, *p* < 0.01). Among all participants who completed the pre–post comparisons (*n* = 69), the median knowledge score increased from 4 (IQR 4–6) to 10 (IQR 9–10), and the median competency score increased from 8 (IQR 7–8) to 10 (IQR 9–10), with large effect sizes (*r* = 0.85 and *r* = 0.81, respectively). Improvements were observed consistently across both subgroups of LHSs and LHWs (Table 2).

**Table 2:** Overall knowledge and competency scores (*n* = 69^a^)

Participants	Variable	Pre-test Median (IQR)	Post-test Median (IQR)	*Z*-value	*p*-value	Effect size (*r*)^b^
Overall (*n* = 69)	Knowledge	4 (4–6)	10 (9–10)	7.1	<0.01	0.85
	Competency	8 (7–8)	10 (9–10)	6.7	<0.01	0.81
LHSs (*n* = 33)	Knowledge	5 (4–6)	10 (9–10)	4.8	<0.01	0.84
	Competency	9 (7–9)	10 (9–10)	4.6	<0.01	0.80
LHWs (*n* = 36)	Knowledge	4 (4–5)	10 (9–10)	5.2	<0.01	0.87
	Competency	8 (7–8)	10 (9–10)	4.8	<0.01	0.80

IQR, interquartile range; LHS, lady health supervisor; LHW, lady health worker.

a. Paired data was available from 69 participants.

b. Wilcoxon signed-rank test. Effect size calculated as *r* = *Z*/√*N*.

### Item-wise change in knowledge scores

Before training, both LHSs and LHWs had reasonably good understanding of the definition of mental health, effective communication strategies with individuals in distress, symptoms of anxiety and warning signs requiring referral to mental health experts. Overall, LHSs scored higher than LHWs on most knowledge items.

However, both cadres of health workers scored relatively low on decision-making actions when presented with anxiety and depression scenarios, simulating a real-life setting. They scored lowest on items related to protecting the dignity of individuals with mental health concerns and identifying support options for them (Table 3). Post-test score percentages showed marked improvement across all items for both LHWs and LHSs.

**Table 3:** Item-wise knowledge responses by lady health supervisors (LHSs) and lady health workers (LHWs) (*n* = 69^a^)

Questions	LHSs (*n* = 33)		LHWs (*n* = 36)	
	Pre-test *n* (%)	Post-test *n* (%)	Pre-test *n* (%)	Post-test *n* (%)
1. Correct definition of mental health	20 (60.6)	32 (97.0)	21 (58.3)	34 (94.4)
2. Most effective way of communicating with a person experiencing mental health challenges	26 (79.0)	31 (93.9)	29 (80.6)	33 (91.7)
3. Most effective way of promoting respect and dignity of people experiencing mental health challenges	8 (24.2)	28 (84.8)	4 (1.0)	27 (75.0)
4. Most common symptom of anxiety	30 (90.0)	31 (93.9)	28 (77.8)	33 (91.7)
5. Recognition of symptoms of anxiety or depression – (scenario-based)	9 (27.3)	25 (75.8)	10 (27.8)	30 (83.3)
6. Core symptom of depression	10 (30.3)	31 (93.9)	17 (47.2)	34 (94.4)
7. Symptom complex best fitting an episode of depression	4 (12.1)	29 (87.9)	11 (30.6)	32 (88.9)
8. Best psychosocial intervention for someone experiencing mental health challenges	17 (51.5)	30 (90.9)	12 (33.3)	33 (91.7)
9. Best option to help a person experiencing mental health challenges – (scenario-based)	4 (12.1)	31 (93.9)	1 (2.8)	33 (91.7)
10. Recognition of specific danger sign requiring referral to mental health specialist – (scenario-based)	25 (76.0)	31 (93.9)	16 (44.4)	35 (97.2)

a. Paired data available from 69 participants.

### Item-wise change in competency scores

The skill assessment form evaluated participants’ competencies across three domains: effective communication with individuals experiencing mental health challenges, provision of psychosocial interventions and referral to specialist mental health services. Item-wise scores of participants before and after the training are shown in Table 4. This indicates that LHWs showed better recognition of effective communication methodologies, whereas LHSs demonstrated stronger comprehension of psychosocial counselling and referral skills at baseline.

**Table 4:** Item-wise competency responses by lady health supervisors (LHSs) and lady health workers (LHWs) (*n* = 69^a^)

Competencies	LHSs (*n* = 33)		LHWs (*n* = 36)	
	Pre-test *n* (%)	Post-test *n* (%)	Pre-test *n* (%)	Post-test *n* (%)
Use of effective communication skills by LHSs and LHWs				
1. Involved the person seeking help in all aspects of identification of symptoms and counselling	30 (90.0)	33 (100.0)	35 (97.2)	36 (100.0)
2. Actively listened to the person seeking help for mental health concerns	30 (90.0)	33 (100.0)	34 (94.4)	36 (100.0)
3. Were friendly, respectful and non-judgemental at all times in interactions with a person experiencing mental health challenges	30 (90.0)	33 (100.0)	34 (94.4)	36 (100.0)
4. Used good verbal communication skills in interactions with a person experiencing mental health challenges	29 (87.9)	33 (100.0)	34 (94.4)	36 (100.0)
5. Responded with sensitivity when people experiencing mental health challenges disclosed difficult experiences	30 (90.0)	33 (100.0)	34 (94.4)	36 (100.0)
6. Protected the confidentiality and consent of people experiencing mental health challenges	23 (69.7)	33 (100.0)	32 (88.9)	36 (100.0)
Provide psychosocial interventions				
7. Performed psychoeducation	23 (69.7)	32 (97.0)	22 (61.1)	33 (91.7)
8. Addressed psychosocial stressors to reduce stress as appropriate for the person experiencing mental health challenges	23 (69.7)	32 (97.0)	24 (66.7)	34 (94.4)
9. Promoted functioning in daily activities, as appropriate for the person with anxiety or depression	26 (78.8)	32 (97.0)	19 (52.8)	33 (91.7)
Refer to mental health specialists’ services				
10. Knew when to refer to a specialist as appropriate and available	23 (69.7)	32 (97.0)	22 (61.1)	34 (94.4)

a. Paired data available from 69 participants.

## Discussion

This study presents one of the first documented efforts to adapt the WHO mhGAP-IG 2.0 into local language for low-literacy, non-specialist community workers of the government’s LHW-P in Pakistan. Our findings show that a contextualised training programme can enhance knowledge and skills of LHWs and LHSs, working within the community to recognise anxiety and depression symptoms, identify appropriate psychosocial counselling, recognise danger signs and determine whether referral to more formal mental health services is required.

Although our study shares similarities with other LMIC initiatives – such as those in Malawi,^24^ Uganda,^26^ Nepal^27^ and Bangladesh,^28^ which have demonstrated the efficacy of mhGAP trainings – the unique characteristics of our LHWs and LHSs sets them apart from participants in previous programmes. Notably, our trainees are distinct from the more formally trained healthcare professionals, such as nurses, doctors and psychosocial workers, who have been the focus of previous studies. The LHWs and LHSs in our study have limited literacy, with a maximum of 8 years of education and no prior mental health background. As government-employed, women CHWs, they provide doorstep primary healthcare services, primarily focusing on MNCH in rural Pakistan. This context differs significantly from other mhGAP training programmes, which have often targeted more educated, facility-based healthcare providers with some mental health training background and have frequently included both genders.

The mhGAP-IG 2.0 is a standardised tool, and adaptation requires careful consideration of the local context, including physical, organisational, institutional and legislative structures that shape healthcare delivery, as well as available resources and workforce capacities.^29,30^ Although a universally accepted approach to mhGAP-IG adaptation is still evolving,^31^ a systematic review of mhGAP implementation across LMICs has emphasised the importance of flexible delivery and local ownership, highlighting the need for adaptations aligned with country-specific health systems, human resources and cultural norms.^32^ In our study, we contextually tailored the content to the roles, educational backgrounds and skill levels of CHWs. In the absence of a formal mental healthcare system in Pakistan, this adaptation also involved identifying and mapping existing referral facilities in the vicinity. The CHWs receiving this contextualised training were trained to refer individuals to local healthcare facilities, which, although not necessarily specialised, are often the most accessible option. This adaptation effectively embedded practical referral pathways into the training, enhancing the workers’ ability to provide meaningful support within the local healthcare landscape.

In addition to structural factors, social determinants such as gender, education and rural residence may shape both CHW experiences and community engagement with the mental health services. These intersectional factors can influence how interventions like the mhGAP are delivered, received and trusted within the community.^33,34^ Although the mhGAP aims to offer a globally applicable framework, its effective use in rural Pakistan benefits from deliberate cultural and linguistic adaptations. Our approach incorporated local idioms of distress, participatory design and context-specific help-seeking behaviours, enhancing linguistic accessibility and community acceptability.^34^ This supported implementation and fostered local ownership.

A study conducted in Tunisia, which tailored mhGAP training to their local context, also highlighted that cultural adaptation should be a central principle in training and implementation.^35^ Users of the mhGAP guide may select and adapt a subset of its priority conditions and interventions based on local prevalence patterns and the availability of resources.^10^ There is growing advocacy that non-specialist health providers, even those without prior mental health training or with limited formal education, can successfully assess patient needs and offer basic support when trained using culturally and contextually validated materials.^36^

Despite initial concerns about adding to LHWs’ workload, the training was well received, with high attendance and completion rates. This can be attributed to the ownership, endorsement and support from the Department of Health, Government of Sindh, which alleviated concerns about competing responsibilities and allowed CHWs to dedicate time to the training. This contrasts with other mhGAP training initiatives, which have struggled with recruitment and engagement, often citing lack of interest or competing priorities as barriers.^37^

The significant improvement in CHWs’ knowledge scores following the mPareshan training is consistent with findings from other pre–post design studies evaluating mhGAP-based training among non-specialist mental health workers.^38^ Item-wise analysis of knowledge scores in our study revealed that CHWs scored low on knowledge questions that required practical application of symptoms of anxiety and depression. This suggests challenges in translating knowledge into real-world practice.

Knowledge scores were also low in recommendation of psychosocial interventions and the need for appropriate referrals. These scores improved substantially post training. Similar findings were observed in a study conducted in Malawi, where allied health workers showed notable gains in knowledge scores after a 2-day mhGAP-informed training.^24^

Both LHSs and LHWs demonstrated relatively high baseline knowledge in certain areas, such as effective communication and recognition of common symptoms of anxiety. This may be attributed to their existing training in counselling mothers to allay their anxiety for childhood immunisation and use of contraception. Literature suggests that modest post-training improvements in knowledge may be adequate when baseline knowledge of mental health concepts is already high. Previous mhGAP evaluations report comparable trends, with post-training mean knowledge scores typically increasing by about 5–15%.^9,39^

Clinical competency reflects the practical application of knowledge and management of individuals affected by mental health and often depends on prior exposure to relevant scenarios. This may explain CHWs’ lower baseline performance on scenario-based items in our study. Performance-based role-plays simulate the deployment of knowledge, attitudes and skills in practice. In our study, competency scores improved significantly, consistent with findings from multi-country assessments conducted in Uganda, Liberia and Nepal, which employed similar assessment tools and standardised role-plays.^40^

Nonetheless, contrasting evidence from Nigeria showed that although overall knowledge of participants improved post training, diagnostic accuracy for scenario-based vignette remained low.^41^ Similarly, studies from Ethiopia^42^ and Malawi^43^ reported improvements in health workers’ knowledge and attitudes but persistent gaps in application competencies, as indicated by low detection rates of MNS conditions. This ‘know–do’ gap is consistent with an African study where the majority of non-specialist health workers fared well in communication skills and empathy, but lacked sufficient training in mental health-specific assessment and providing direct care.^40^

These findings suggest that although training programmes can effectively improve knowledge and attitudes, they may not necessarily translate to improved competencies or diagnostic accuracy. This highlights the need for more comprehensive training approaches that incorporate hands-on practice, scenario-based learning and ongoing mentorship to bridge the gap between knowledge and practice.

### Strengths

Although the mhGAP-IG has been implemented in various LMICs, there are fewer documented examples of its systematic adaptation for a government-run, community-based health workers programme in a rural South Asian context. The manual was locally adapted and culturally contextualised, making it suitable for replication in contexts with similar health-system structures.

Mental health is typically neglected in the training curricula of CHWs in Pakistan. As a result, their knowledge and skills in detecting, managing or referring individuals affected by mental health disorders are often inadequate. Despite this limited baseline exposure, our mhGAP-based training proved effective in significantly enhancing our CHWs’ knowledge and competencies in recognising basic symptoms of anxiety and depression, offering psychosocial counselling and initiating appropriate referrals to specialised care when needed.

### Limitations

Comparisons between the findings of this study and those of similar mental health training evaluations conducted elsewhere should be interpreted with caution because of contextual differences. First, we deliberately included active LHWs to ensure that participants of the training were actually involved in home visits and reporting regularly to their supervisors. Without this experience and contextual understanding, comprehension and application of the training content would not have been possible. Although this approach enhanced feasibility and ecological validity, it may limit generalisability to the broader LHW workforce and possibly influence the effectiveness of the training. However, the training’s structured and simplified design makes it suitable for wider rollout among less active LHWs as well, who may demonstrate even greater gains starting from a lower baseline knowledge. Second, the training content, including role-play exercises, were adapted to align with the educational background, literacy levels and work experience of LHWs and LHSs in Pakistan, and was delivered over a limited time frame. Third, the tools used to assess knowledge and competencies were adapted from the original mhGAP materials to fit the routine field activities of LHWs, and may need further adaptation in other settings. Finally, rather than employing a highly trained professional as a co-facilitator, we engaged a community-based individual during role-plays, to simulate a more realistic implementation setting. Although using community members in role-plays may have introduced variability, it also increased ownership and mirrored real-world interactions.

The authors also acknowledge that the use of some terms in the manual may carry unintended value judgements. These terms were used to simplify complex mental health concepts for CHWs with limited literacy and little prior exposure to mental health. As this was the first adaptation of such content for rural primary care settings in Pakistan, the focus was on clarity and relevance. Future adaptations of the manual in other settings can give due consideration to the use of more person-first nomenclature.

In conclusion, our study demonstrates that adapting the WHO mhGAP-IG to the local context and language significantly improves mental health knowledge and skills among CHWs. This contextual adaptation is crucial for integrating mental health into primary healthcare, particularly in resource-constrained settings. Supportive supervision and programme ownership are essential for sustaining and scaling up such initiatives. Our findings suggest that this adapted mPareshan curriculum can be a viable tool for workforce development in mental health. Following the evaluation of this training, the Government of Sindh, Pakistan, endorsed mental health to be part of the LHW-P curriculum.^23,44^ Thus, by contextualising training to local context, we can effectively bridge the mental health treatment gap by empowering these frontline primary healthcare workers as first responders. This approach has the potential for national scalability, and in similar settings. Contextual adaptation, however, is the key to unlocking mental health workforce capacity.

## Data Availability

Data are saved in the Aga Khan University’s repository in the forms of questionnaires and .sav files, which can be made available by the corresponding author, F.R., upon request.
